# The educational and labor market returns to preschool attendance in Austria

**DOI:** 10.1080/00036846.2019.1584368

**Published:** 2019-03-05

**Authors:** Pirmin Fessler, Alyssa Schneebaum

**Affiliations:** a Foreign Research Division, Oesterreichische Nationalbank, Vienna, Austria; b Department of Economics, Vienna University of Economics and Business, Vienna, Austria

**Keywords:** Returns to preschool/kindergarten, early childhood education, education, inequality, I21, H52, J62, I38

## Abstract

Preschool attendance is widely recognized as a key ingredient for later socioeconomic success, mothers’ labor market participation, and leveling the playing field for children from disadvantaged backgrounds. However, the empirical evidence for these claims is still relatively scarce, particularly in Europe. Using data from the 2011 Austrian European Union Statistics of Income and Living Conditions (EU-SILC), we contribute to this literature by studying the effects of having attended preschool for the adult Austrian population. We find strong and positive effects of preschool attendance on later educational attainment, the probability of working full time, hourly wages, and the probability that the mother is in the labor market. Full time workers at the bottom and the top of the distribution benefit less than those in the middle. Women in particular benefit more in terms of years of schooling and the probability of working full time. Other disadvantaged groups (second generation migrants; people with less educated parents) also often benefit more in terms of education and work.

## I. Introduction

Preschool^1^ 1Throughout this paper, we call all forms of schooling before primary school, including kindergarten, ‘preschool.’ is widely discussed as a potential tool to give children the best opportunities for success and to combat socioeconomic inequality. The literature does indeed show that preschool has many positive impacts on individuals in terms of their social, cognitive, and economic development. This paper contributes to the literature on early childhood education and economic inequality by studying the economic and social impacts of preschool attendance in Austria. Using a broad set of econometric methods, we look at the role of preschool attendance in a battery of economic outcomes, for different groups of individuals (men/women; native/migrant; and descendants of high versus low educated parents). In particular, we study the relationship between preschool attendance and (1) educational attainment (total years of schooling and the probability of completing university), (2) labor market outcomes (hourly wages and the probability of working full time), (3) the probability that the respondent’s mother participated in the labor force when the respondent was 14, and (4) wage inequality.

This is the first study to examine the impacts of preschool attendance for people in Austria, making it a contribution to our knowledge of European educational systems and the impact of early childhood education in an institutional context of relatively early tracking ages and low intergenerational educational mobility. The tracking age in Austria and Germany is ten, as compared to 15–16 in the other European countries discussed throughout this paper (France, Spain, the UK, and Norway) (European Commission 2015a, 2015b). In this context, preschool may be particularly helpful for later socioeconomic outcomes, since it could affect the decision about which schooling track to follow and thus the chances for finishing tertiary education and having higher wages. In other words, because preschool attendance can strongly impact the behavior and skills of a student even later, when they are ten years old, it can influence the schooling track onto which the student moves. This latter choice will have severe consequences on the later choices about an academic or a vocational training and later job. Thus, preschool can be decisive for many later outcomes. Moreover, Austria has one of the lowest rates of intergenerational education mobility across Europe (Schneebaum, Rumplmaier, and Altzinger 2015). Here, too, preschool can be decisive in helping children to reach educational and economic achievements beyond what their parents had achieved.

We find that across the board, preschool attendance is a positive force in determining later outcomes at the personal (education, labor force attachment, and wages), familial (mother’s labor force participation), and social (equality in hourly wages) levels. Attending preschool is associated with both a quantitative improvement in educational outcomes, via a 0.4 year increase in the years of schooling completed, and a qualitative improvement, by raising the probability of finishing a higher education degree by 4.6 percentage points. Preschool attendance also increases the chances of working full time by 5.8 percentage points and hourly wages by 7.1 percent, on average. The impacts on years of schooling and the probability of working full time are stronger for women; indeed preschool attendance reduces the gender gap in the probability of working full time by more than a third. This outcome is particularly important in Austria, which has one of the highest rates of part-time employment for women in Europe. Another significant gender effect of preschool attendance is that the probability of a mother working when her child is 14 is 8.4 percentage points higher when the child had attended preschool. Finally, preschool has the strongest wage impact for people in the middle of the income distribution, and it lowers inequality in the upper half of the income distribution.

Before turning to the quantitative effects of preschool that have so far been identified for other European countries, we first consider the mechanisms through which preschool attendance might lead to positive outcomes. Carneiro and Heckman (2003) and Cunha et al. (2006) make clear that *early* investment in education (as in attending preschool) is particularly helpful in developing both cognitive and behavioral skills, because gains in IQ are easiest and highest at younger ages and because earlier investment means that there is more time to reap the benefits of the investments. But how do the effects come into place? An analysis by Heckman, Pinto, and Savelyev (2013) suggests that it is the preschool program’s impact on academic motivation (measured by indices for showing initiative, being alert and interested in school work, and not being hesitant to try or giving up easily) and externalizing behavior (disrupting classroom procedures; swearing or using obscene words; stealing; lying or cheating; influencing others towards trouble-making; acting aggressively towards peers; teasing or provoking students) in particular that positively impact cognitive and personality skills. In the present paper, we study long-term impacts of preschool attendance. As Chetty et al. (2011) say, evidence on the long-term impacts of early childhood education ‘remain scarce because of a lack of data linking childhood education and outcomes in adulthood’ (p. 1594). They show that the mechanism through which the positive effects of preschool attendance (on later earnings, educational attainment, home ownership, and retirement savings) is likely the non-cognitive skills developed while in preschool. These helpful skills held by preschool attendees, measured based on what teachers said about the students in their classes, were effort, initiative, engagement in class, and whether the student values school. Thus, preschool attendance seems to be effective by positively impacting motivation and the ability to learn in the classroom environment in particular.

Turning to the quantitative effects of preschool attendance, there is a large literature on the impact of early childhood education on one’s later outcomes, particularly for the U.S. Although dated, Currie (2001) gives an excellent review of the (primarily U.S.) literature, showing that preschool attendance leads to a wide range of positive effects, from higher IQ, better scores on academic tests, and higher graduation rates, to lower probabilities of being held back a grade while in school or being unemployed, on welfare, in jail, or pregnant as a teen. Given that the present study focuses in particular on the impact of preschool attendance on educational attainment, labor force attachment and wages earned, mother’s labor force participation, and wage inequality in Austria, we restrict our review of this literature to studies from other European countries with similar outcome measures. While several studies look at the benefits of preschool to children’s general social, emotional, and cognitive development in various European countries and for heterogeneous groups (e.g. Felfe and Lalive (2013) for Germany, Esping-Andersen et al. (2012) and Bauchmüller, Gørtz, and Rasmussen (2014) in Denmark, Leuven et al. (2010) in the Netherlands, and Fredriksson et al. (2010) for Sweden), we focus on literature which has similar individual-level outcome variables to our own.

Turning first to the effect of preschool attendance on a person’s later educational attainment and earnings, the literature fairly consistently reports positive impacts, albeit with differing degrees of strength for people in various socio-demographic groups. In studying the effects of a reform expanding preschool availability in Norway, for example, Havnes and Mogstad (2011) find that the expansion resulted in 0.35 more years of schooling, on average; an increase of college attendance rates of 6.8 percentage points; and a decrease in high school drop-out rates of 5.8 percentage points. Most of these positive effects are driven by exceptionally strong results for children with less educated mothers, and for girls, who are about seven percentage points less likely to become low earners if they received the preschool treatment. The same authors show in a later study that attending preschool leads to higher earnings, particularly for women and for people from a lower socioeconomic background. Indeed girls who were exposed to the preschool reform had higher earnings than girls who did not, while boys who got a preschool education actually had lower earnings than boys without a preschool education (although these findings are not statistically significant) (Havnes and Mogstad 2015).

Dumas and Lefranc (2010) study the effects of reforms which expanded preschool enrollment in France in the 1960s and 70s, finding that an additional year of preschool reduced the probability of needing to repeat a grade later on by two percentage points and increased the probability of graduating from high school by almost three percentage points. This result is driven by positive effects found for people from lower- and middle-class backgrounds. (Gupta and Simonsen (2010) also show that in Denmark, public childcare was particularly beneficial for children from disadvantaged backgrounds.) Moreover, the authors find that starting preschool a year earlier increases monthly wages as an adult by about three percent. These results hold only for people from lower and middle class backgrounds.

In a similar study design, Felfe et al. (2015) look at the effect of an expansion of high-quality preschool spots for 3-year-olds in Spain in the early 1990s. The authors find that 15-year-olds who lived in Spanish states with the largest increases in preschool enrollment after the reform had higher scores on the math PISA test than students in states with lower levels of preschool enrollment expansion. As in the studies discussed above, the positive effects are strongest for girls and children of parents with lower educational attainment.

Two European studies of the educational effects of preschool attendance use a similar estimation framework as ours, which is described in the next section. First, with data from the U.K., Goodman and Sianesi (2005) show that pre-compulsory school attendance increases the probability of obtaining a degree or other higher education qualification for women (but not men), though the magnitude of this effect is not reported.^2^ 2Similarly, in the U.S., Anderson (2008) found that the Perry Preschool, Abecedarian, and the Early Training Project Programs had a statistically significant positive relationship with the cognitive development and later economic success of girls, but not boys. These findings were in contrast to earlier studies, because Anderson (2008) corrected for the potential for rare events in the multiple inference framework in earlier analyses which had found positive results for boys only. Further, Goodman and Sianesi (2005) find that preschool attendance is related to an increase of three percent in wages for women (but not men) up through age 33. When respondents were surveyed at age 42, the positive effect of preschool on earnings had disappeared. Second, with an analysis of a rich dataset (the German Socio-Economic Panel) from West Germany, Katharina, Büchel, and Wagner (2003) find that attending preschool leads to a tremendous increase in the probability of being assigned to an academic school track for second generation migrant children, but not for native German children.

Aside from later educational attainment and earnings, we are also interested in assessing if preschool attendance has an impact on one’s mother’s labor force participation. The authors know of only three papers which study this effect in European countries. Havnes and Mogstad (2011) and Black et al. (2014) find no net effect of preschool on mothers’ labor force participation in Norway and Felfe et al. (2015) finds no effect in Spain. The seminal paper in the U.S. literature, though, suggests that at least in some states (those in which there are data and policy reforms which allow an experimental design), preschool boosts the probability of a mother working for married mothers (by about seven percent) and single mothers whose youngest child is the one in preschool (by about six percent) (Gelbach 2002). Cascio (2009) confirms these findings for single mothers (the probability of having worked last week increases by 7.5 percent), but finds less strong effects for married women. While an early analysis found that there was no effect of the implementation of universal preschool on mother’s labor force participation (Fitzpatrick 2010), the same author later used a different estimation technique and could confirm that the implementation of free preschool increases the probability of working of (only) single mothers whose youngest child is preschool age by between 15 and 20 percentage points (Fitzpatrick 2012). Preschool and preschool availability have also been shown to have a positive impact on mothers’ labor force participation in Argentina (seven to 14 percentage point increase in the probability of working) (Berlinski and Galiani 2007), Québec, Canada (6.5 percentage points for mothers with a high school diploma; 7.9 percentage points for all mothers) (Lefebvre and Merrigan 2008), and on Arab mothers in Israel (seven percentage points) (Schlosser 2005).

Finally, three European papers look explicitly at the impact of expanded preschool access on wage inequality and find an equalizing effect of preschool on the wage distribution. Havnes and Mogstad (2011) show that both boys and girls who attended preschool are 2.2 percentage points less likely to become top earners, while girls are seven percentage points less likely to become low earners. Preschool attendance also increases the probability of girls becoming average earners by almost 8.5 percentage points. The same authors later show that attending preschool increases the earnings of people from a low socioeconomic background by almost three percent and decreases them for people from a high socioeconomic background by about two percent (Havnes and Mogstad 2015, Table 2). Further, the wage effects of preschool attendance are highest for people from lower earnings households, with a peak in the effect at about the $11^{th}$ percentile of the household earning distribution and negative effects after the $82^{nd}$ percentile. Finally, the analysis in Dumas and Lefranc (2010) reveals that preschool increases monthly earnings for people whose parents were in social group one (farmers and manual workers) and social group two (non-manual workers), while decreasing wages for those whose parents were higher-grade professionals.

**Table 1.** Descriptive statistics.

	All	Preschool	No Preschool
Preschool	0.60	1.00	0.00
	(0.007)		
Mean age	43.0	39.8	47.9
	(0.142)	(0.183)	(0.168)
Age 25–34	0.23	0.34	0.06
	(0.006)	(0.009)	(0.006)
Age 35–44	0.29	0.33	0.24
	(0.006)	(0.009)	(0.010)
Age 45–59	0.48	0.33	0.71
	(0.007)	(0.009)	(0.011)
Female	0.50	0.49	0.50
	(0.007)	(0.009)	(0.012)
Primary education	0.13	0.09	0.19
	(0.005)	(0.005)	(0.010)
Lower secondary education	0.42	0.38	0.49
	(0.007)	(0.009)	(0.012)
Upper secondary education	0.30	0.34	0.24
	(0.007)	(0.009)	(0.010)
Tertiary education	0.15	0.19	0.09
	(0.005)	(0.007)	(0.006)
Years of schooling	11.49	11.93	10.83
	(0.036)	(0.049)	(0.049)
Second Generation Migrant	0.02	0.02	0.02
	(0.002)	(0.003)	(0.003)
Number of Observations	5,679	3,429	2,250

*Notes*: This table shows the means of the main variables used in the sample. Standard errors are given in parentheses. *Source*: Authors’ calculations on EU-SILC 2011.

**Table 2.** Effects of preschool attendance on years of schooling.

	Est	(s.e.)	Est	(s.e.)	Est	(s.e.)
Preschool attendance	1.174	(0.068)	0.426	(0.073)	0.382	(0.084)
Intercept	10.935	(0.048)	11.385	(0.052)	11.424	(0.070)
Age			0.120	(0.030)	0.073	(0.064)
Age squared			−0.001	(0.000)	−0.001	(0.001)
Female			0.130	(0.062)	−0.030	(0.087)
Father ed. 2			0.163	(0.072)	0.102	(0.094)
Father ed. 3			1.585	(0.141)	1.723	(0.282)
Father ed. 4			2.503	(0.196)	3.456	(0.446)
Mother ed. 2			0.482	(0.089)	0.442	(0.153)
Mother ed. 3			1.377	(0.118)	1.040	(0.222)
Mother ed. 4			2.184	(0.260)	2.241	(0.616)
Parent immigrant			−0.578	(0.223)	−0.824	(0.354)
PSAxAge					0.090	(0.075)
PSAxAge squared					−0.001	(0.001)
PSAxFemale					0.264	(0.122)
PSAxFather Ed. 2					0.112	(0.142)
PSAxFather Ed. 3					−0.165	(0.329)
PSAxFather Ed. 4					−1.116	(0.499)
PSAxMother Ed. 2					0.022	(0.189)
PSAxMother Ed. 3					0.402	(0.263)
PSAxMother Ed. 4					−0.009	(0.677)
PSAxParent immigrant					0.322	(0.460)
Linear Controls			Yes		Yes	
Heterogenous TE					Yes	
N	5345		5345		5345	

*Notes*: This table shows the average treatment effect of preschool attendance (PSA) on years of schooling. Demeaned variables are used for all covariates and interactions. Regional dummies and interactions were included as controls (not shown). *Source*: Authors’ calculations on EU-SILC 2011.

In the next section of this paper, we describe our methodology for assessing the impact of preschool attendance on our outcomes of interest. In section 3 we present the data we use and section 4 gives the results of our analysis.

## II. Estimation strategy

In this section, we describe our strategy to estimate the effects of preschool attendance on later outcomes. Many, though not all, of the studies discussed above identify preschool effects by assessing the results of an exogenous policy change that expanded access to preschool. Using such exogenous variation to assess the effect of attending preschool leads to estimates that can certainly be interpreted as causal effects with near-perfect internal validity; they deliver precise and unbiased estimates of the effect of preschool for the small subgroup they study. However, this approach is not quite appropriate for the goals of this paper. Here, we seek to assess the impact of attending preschool for the entire working-age population and over the full distribution of wages in the population. Using a natural experiment such as a school reform would only allow us to identify causal effects for a small subgroup comprising a few cohorts.

We thus instead rely on a rich dataset that contains not only information on an individual’s preschool attendance and demographic characteristics, but also on their family and economic background, representative of the Austrian population. This approach seems to us appropriate, given that we study the impact of preschool attendance on the whole population, not just on a selected sub-population (in particular, a birth cohort) that experienced a policy change). To estimate the (predictive) effect of preschool enrollment on later economic outcomes, we draw on the recent microeconometric literature on causal effects, program evaluation, and decomposition methods. The workhorse for our analysis of preschool effects is a fully integrated linear model with a functional form allowing for heterogeneous treatment effects and straightforward interpretation, which was proposed in Imbens and Rubin (2015). For our analyses beyond the average effect we also use the propensity score (Rosenbaum and Rubin 1983) and re-centered influence function regressions (Fortin, Lemieux, and Firpo 2009) to estimate the returns to preschool attendance on later earnings. Most of these decomposition techniques are summarized in, for example, Fortin, Lemieux, and Firpo (2011) and discussed in depth in Appendix B. We also include estimates of the mediation effects in our prediction of the effect of preschool on wages, where we use standard approaches discussed in Pearl (2009) or VanderWeele (2015).

The key challenge to our estimation technique is the threat of selection bias. In particular, parents who send their children to preschool may be exactly the parents who are most likely to value education. Thus, children who go to preschool benefit not only from preschool itself, but also from their parents’ appreciation for education. To assess the impact of attending preschool, we need to separate the effects of preschool attendance itself from the effects of the other characteristics in order to identify the relationship between preschool and later outcomes. It is the choice of background characteristics that is critical in purging selection bias from our estimates. The better we are able to model selection into preschool, the closer our (predictive) effects will be to causal effects. The ‘effects’ we estimate are thus not to be interpreted as pure causal effects but are, as we argue below, reasonably well purged of selection bias to illustrate the direction and mechanism of the distributional (predictive) effects. Studying the relationship between preschool attendance and later outcomes while eliminating potential selection bias by controlling for background characteristics that likely determine selection into preschool is the same empirical strategy also employed by Goodman and Sianesi (2005) for the UK and Berlinski, Galiani, and Manacorda (2008) for Uruguay. We discuss the choice of background variables we choose to help eliminate selection bias in detail in the next section.

Different techniques to control for selection bias such as matching and regression with controls are valid under the same identifying assumption of conditional independence (see a discussion on identification in Appendix A). In our analyses of the relationship between preschool attendance and later socioeconomic outcomes, we use a regression-based approach to study both average effects and effects across the distribution of the outcome. First and foremost, we use simple observed differences in outcomes for those with and without preschool attendance as a benchmark measure, which we estimate using standard ordinary least squares, regressing the economic outcome of interest on a preschool dummy:

(1) $$Y_{i}=\alpha +\beta \cdot T_{i}+\varepsilon_{i}.$$

We then gradually increase control over selection bias by adding in the demeaned background characteristics $\left(X_{i}-\overset{ˉ}{X}\right)$ to the OLS model. Note that demeaning here does not change the coefficients, aside from centering the intercept.

(2) $$Y_{i}=\alpha +\beta \cdot T_{i}+(X_{i}-\overset{ˉ}{X})\gamma +\varepsilon_{i}.$$

Finally, to allow the treatment effect to be heterogenous across different individuals, we increase flexibility by interacting the preschool dummy with all covariates in X. This model allows the preschool effect to be different for individuals with different characteristics. As Imbens and Rubin (2015) propose, we include the covariates in deviations from the sample average, so that the estimated coefficient on the treatment indicator β can be interpreted as an estimate for the average treatment effect of the treatment in the population. Implicitly, this specification allows us to have separate slope coefficients for treated and control regression functions.

(3) $$Y_{i}=\alpha +\beta \cdot T_{i}+(X_{i}-\overset{ˉ}{X})\gamma +T_{i}(X_{i}-\overset{ˉ}{X})\theta +\varepsilon_{i}$$

In equations (1) through (3), $\varepsilon_{i}$ denotes an error term with mean zero and $\sigma^{2}$ variance.

Aside from the preschool effects at the mean, we use two methods to investigate the impact of preschool attendance across the full distribution of the economic outcomes P(Y) and their related measures ν(P(Y)); in this case, the outcome of interest is hourly wages. First, we use the propensity score to balance the covariates of individuals with and without preschool attainment in order to construct counterfactual populations, as proposed in Rosenbaum and Rubin (1983) and DiNardo, Fortin, and Lemieux (1996). and dinardo1996. Second, as a robustness check, we use more flexible re-centered influence function (RIF) regressions to study the preschool effect across the full distribution of wages. A re-centered influence function is similar to a standard regression, except that the dependent variable is replaced by the re-centered influence function of the statistic of interest (see Fortin, Lemieux, and Firpo 2009). The re-centered influence function approach we specify is as flexible as equation 3, as it also allows for heterogenous treatment effects across all covariates. In Appendix B we provide a short discussion of both of these methods.

In the next section we discuss the dataset used in our analysis. We give considerable attention to the background characteristics in X, as they are key in reducing selection bias in our estimates as much as possible.

## III. Data

We employ data from the 2011 Austrian European Union Statistics on Income and Living Conditions (EU-SILC) dataset (Statistik Austria, 2014) for this analysis. The data provide information on demographic, economic, and family background characteristics of 13,933 individuals.

Our main variable of interest is preschool attendance. The relevant survey question asked respondents if they had attended kindergarten or preschool (*‘Kindergarten’ or ‘Vorschule’*) (answers were either simply yes or no, meaning that there is no information on the length of preschool attendance or the characteristics of the institution visited). Only individuals aged 25–59 were given the special module asking about preschool, which is an unproblematic restriction because we are mainly interested in labor market outcomes and this is standard working age in Austria. We further dropped all individuals who were not born in Austria or who moved to Austria before the age of four (1,085 observations), in order to avoid conflating the effects of preschool attendance in other countries with those in Austria. Of the remaining 5,707 observations, we drop 16 who stated that they did not live in Austria at age of 14, and 12 further individuals who did not provide information on their preschool attendance. The final sample comprises 5,679 individuals.

The most important background characteristics in our data are the educational attainment of the mother and the father. Parental education explains a large part of an individual’s later outcomes, since it is highly correlated with parental wealth, income, and health, which all affect descendant outcomes in a positive way (Haveman and Wolfe 1995). Parental education is thus an important and credible proxy for social background. Highly educated parents are also more likely to value schooling and be attached to the labor market, which would make them more likely to enroll their children in preschool. At the same time, parental education is typically fixed well before the decision to send children to preschool. It is thus an ideal candidate for a covariate determining treatment assignment.

In addition, we include information on the financial situation of the household at age 14 as assessed by survey respondents as adults, as a proxy for the situation when their parents were making the decision of whether or not to send the child to preschool. While we control for the financial situation of the household, preschool is heavily subsidized in Austria, meaning that social transfers make preschool attendance affordable for children from any household. In this sense, concerns about selection into preschool attendance based on economic circumstances are largely mitigated by the institutional framework of the country under study.

Other important covariates in our data are age (and its square), gender, a dummy variable for being a second-generation migrant, and regional dummies for the nine provinces of Austria in which the respondent lived at age 14, as a proxy for the region in which s/he likely lived before the time of the preschool decision. The region dummy is important because the cost and conditions of the preschool (i.e. the maximum number of children in a group, hours of operation, and the number of weeks per year the institution can be closed) vary based on regulations at the regional level (Baierl and Kaindl 2011). Within our regional controls, therefore, there is no differences in costs or conditions of state-provided preschools.

While the covariates in our data can go a long way in predicting selection into preschool attendance, and the institutional framework in Austria make it highly unlikely that parents who want to send their children to preschool are unable to do so, we are still cautious about calling our estimates ‘causal effects.’ Instead we aim at presenting predictive effects, which are likely to be reasonably purged of selection bias to ensure feasible estimates for the full population. The approach we take in this paper thus offers a balance between the internal validity of estimates derived from analyzing a policy reform, and the external validity of estimates for the entire population. Given the data at hand and the institutional framework in our country under study, this approach is the best to meet our goals.

Table 1 presents main descriptive statistics for our sample. About 60% of the the adult Austrian population aged 25 to 59 attended preschool as a child. The share of individuals enrolled in preschool has slightly increased over time. Among the population who did not attend preschool, only about 6% are younger than 35, while this youngest cohort represents 23% of the population but comprises 34% of all people who attended preschool. Males and females were almost equally likely to attend preschool, but the distribution of preschool attendance varies greatly by (later) educational attainment. Overall, about 15% of the population has a tertiary education, but 19% of preschool goers and only 9% of people without preschool have a tertiary education. Accordingly, average years of schooling is lower in the population without preschool attainment. Two percent of our sample is second generation migrants. The small share of second generation migrants is due to the fact that the major recent migration waves into Austria occurred in the 1960s and 1970s, as ‘guest workers’ came from Yugoslavia and Turkey, and in the 1990s, because of the Yugoslavian war. The large majority of the children of these migrants will have been born after the cutoff dates for participation in this module of the SILC survey.^3^ 3The respondents in our sample were 25–59 in 2010 (and thus born between 1951–1985).


## IV. Empirical results

### Effects on educational attainment

We begin the presentation of our results with a look at the effect of preschool attendance on the later educational attainment of the individuals in our sample. In all specifications, the vector of control variables X comprises variables for mother’s educational attainment, father’s educational attainment, age, age squared, gender, dummy variables for the Austrian region of residence at age 14, dummy variables for the financial situation of the household at age 14, and a dummy for birth in Austria. In Table 2 and onwards, ‘PSA’ is an abbreviation for ‘preschool attendance’ and parental education classes are shown as ‘father/mother educ. 2/3/4’, where category 2 is lower secondary, 3 is upper secondary, and 4 is tertiary (the omitted category is maximum primary education). The first two columns in Table 2 give the results of the unconditional difference in years of schooling^4^ 4Years of schooling is the minimum years of schooling necessary to achieve the level of education reported by the respondent. for those who did versus those who did not attend preschool (equation 1), followed by OLS with linear demeaned controls in the middle two columns (equation 2) and a fully interacted linear model on demeaned controls (equation 3) in the last two columns. The raw difference shows that people who attended preschool have an average of 1.17 more years of education. The average treatment effect of preschool attendance on years of schooling is estimated at 0.43 in the model with linear controls, while the one allowing for heterogenous treatment effects – our preferred specification – lies at 0.38 additional years of school (or, put differently, 0.15 standard deviations of years of schooling). Decomposing the total difference in years of schooling (1.17) into the (predictive) effect of preschool attendance (0.4), we can say that about one-third of the raw difference is the predictive effect purged of the selection bias we can eliminate with our variables in X.

The literature discussed in section 1 finds that the benefits of attending preschool differs across groups. The results in Table 2 show that the impact of attending preschool on years of schooling is larger for females (an additional 0.26 years) and migrants (although the latter estimates are economically but not statistically significant at conventional levels), and smaller for those with more highly educated father. The lower returns for children of highly educated parents is not surprising, since these children have more resources at their disposal throughout their lives; kindergarten will thus help them relatively less.

While the effect of preschool attendance on years of schooling is a quantitatively important .4 years added, Table 3 shows that this extra time in school also brings a qualitative improvement to educational attainment. Here we show average marginal effects based on logit models using the same flexible form allowing for heterogenous treatment effects when predicting the probability of completing tertiary education.^5^ 5Tertiary education is defined here as having graduated from university or a *Fachhochschule*. Attending preschool makes one 4.6 percentage points more likely to complete a degree. This result is highly economically and statistically significant. Moreover, the effect is stronger for women and especially second generation migrants, although these differences are not statistically significant.

**Table 3.** Effects of preschool attendance on the probability of completing higher education.

	Est	(s.e.)	Est	(s.e.)	Est	(s.e.)
Preschool attendance	0.125	(0.011)	0.040	(0.011)	0.046	(0.015)
Intercept	−0.302	(0.008)	−0.237	(0.007)	−0.241	(0.012)
Age			0.016	(0.004)	0.015	(0.013)
Age squared			−0.000	(0.000)	−0.000	(0.000)
Female			0.005	(0.009)	−0.005	(0.018)
Father ed. 2			0.017	(0.012)	−0.005	(0.022)
Father ed. 3			0.132	(0.015)	0.157	(0.029)
Father ed. 4			0.203	(0.018)	0.266	(0.044)
Mother ed. 2			0.035	(0.013)	0.050	(0.026)
Mother ed. 3			0.110	(0.013)	0.107	(0.026)
Mother ed. 4			0.190	(0.025)	0.267	(0.085)
Parent immigrant			−0.047	(0.044)	−0.146	(0.136)
PSAxAge					0.005	(0.014)
PSAxAge squared					−0.000	(0.000)
PSAxFemale					0.014	(0.021)
PSAxFather ed. 2					0.028	(0.027)
PSAxFather ed. 3					−0.032	(0.034)
PSAxFather ed. 4					−0.074	(0.049)
PSAxMother ed. 2					−0.026	(0.030)
PSAxMother ed. 3					−0.003	(0.030)
PSAxMother ed. 4					−0.087	(0.089)
PSAxParent immigrant					0.119	(0.144)
Linear Controls			Yes		Yes	
Heterogenous TE					Yes	
N	5345		5345		5345	

*Notes*: This table shows the average treatment effect of preschool attendance (PSA) on the probability of completing tertiary education. Demeaned variables are used for all covariates and interactions. Regional dummies and interactions were included as controls (not shown). *Source*: Authors’ calculations on EU-SILC 2011.

In sum, preschool attendance increases both quantitative (years of schooling) and qualitative (probability of completing higher education) educational attainment. These results are in line with, although slightly higher than, the findings in the literature: the increase of years of schooling of .38 years compares to .35 years in the UK Havnes and Mogstad (2011), and the 4.6 percentage point increase in the probability of completing a higher education degree is higher than the 1.5 percentage point increase found in Goodman and Sianesi (2005). The fact that the preschool effects are stronger in Austria could be explained by the relatively low share of people with a tertiary degree (OECD 2016) and the earlier tracking age (European Commission 2015a) compared to Norway and the UK.

### Effects on labor market outcomes

Table 4 shows that the effects of preschool attendance on current labor force participation. Attending preschool increases the likelihood of working full time by 5.8 percentage points. This effect is especially pronounced for women, who are an *additional* 9.7 percentage points more likely to work full time if they attended preschool as children. The raw, overall gender gap in the probability of working full time is 39.7 percentage points (see the coefficient on the female dummy variable in the full model). Importantly, the total preschool effect for women (5.8 + 9.7 = 15.5 percentage points) is more than a third of that gap. In this sense, preschool attendance is remarkably effective in promoting women’s presence in the labor force. As in Goodman and Sianesi (2005), the effect of preschool attendance on the probability of working is stronger for younger people. The negative coefficient on the preschool and age interaction shows that preschool is less important in predicting the probability of working full time for older people.

**Table 4.** Effects of preschool attendance on working full time.

	Est	(s.e.)	Est	(s.e.)	Est	(s.e.)
Preschool attendance	0.049	(0.014)	0.035	(0.016)	0.058	(0.019)
Intercept	−0.040	(0.011)	−0.032	(0.012)	−0.053	(0.016)
Age			−0.013	(0.007)	0.032	(0.015)
Age squared			0.000	(0.000)	−0.000	(0.000)
Female			−0.339	(0.009)	−0.397	(0.019)
Father ed. 2			0.053	(0.016)	0.057	(0.024)
Father ed. 3			0.034	(0.026)	0.112	(0.055)
Father ed. 4			0.050	(0.034)	0.091	(0.098)
Mother ed. 2			−0.002	(0.018)	−0.036	(0.036)
Mother ed. 3			−0.009	(0.021)	−0.019	(0.043)
Mother ed. 4			−0.059	(0.050)	−0.117	(0.196)
Parent immigrant			−0.077	(0.053)	−0.004	(0.088)
PSAxAge					−0.058	(0.018)
PSAxAge squared					0.001	(0.000)
PSAxFemale					0.097	(0.027)
PSAxFather ed. 2					−0.006	(0.032)
PSAxFather ed. 3					−0.099	(0.063)
PSAxFather ed. 4					−0.047	(0.105)
PSAxMother ed. 2					0.046	(0.042)
PSAxMother ed. 3					0.015	(0.049)
PSAxMother ed. 4					0.070	(0.202)
PSAxParent immigrant					−0.094	(0.109)
Linear Controls			Yes		Yes	
Heterogenous TE					Yes	
N	5019		5019		5019	

*Notes*: This table shows the average treatment effect of preschool attendance (PSA) on the probability of working full time. Demeaned variables are used for all covariates and interactions. Regional dummies as well as dummies for the financial situation of the household and interactions were included as controls (not shown). People who report being retired are excluded from the sample. *Source*: Authors’ calculations on EU-SILC 2011.

The next set of empirical exercises calculate the effect of preschool attendance on gross hourly wages. We first calculate Mincerian returns to education for our data. The first two columns in Table 5 show that for all employees in our sample, an additional year of schooling increases hourly wages by 6.4%. Adding experience and its square in the next two columns brings this figure closer to eight percent. These findings are in line with other literature on the returns to education in Austria (Fersterer and Winter-Ebmer 2003). The next two columns add a dummy variable for preschool attendance and show that adding preschool to the model hardly changes the returns to years of schooling, but that the returns to preschool are 6.7%, a rate comparable to an additional year of schooling. The model in the last two columns also adds a control for parental education, and reveals that despite this addition, the preschool effect is still at 5.7 percent and the wage returns to education are at 7.6^6^ 6This equals 0.19 standard deviations of log hourly gross wages. percent.

**Table 5.** Mincerian returns to education and preschool attendance.

	Est	(s.e.)	Est	(s.e.)	Est	(s.e.)	Est	(s.e.)
Years of schooling	0.064	(0.003)	0.078	(0.003)	0.077	(0.003)	0.076	(0.003)
Experience			0.034	(0.003)	0.035	(0.003)	0.035	(0.003)
Experience squared			−0.001	(0.000)	−0.001	(0.000)	−0.000	(0.000)
Preschool attendance					0.067	(0.015)	0.057	(0.015)
Classical Mincer			Yes		Yes		Yes	
Parental Education							Yes	
N	2695		2695		2695		2695	

*Notes*: This table shows classical wage regressions (log hourly gross earnings) for all employees, adding preschool attendance as well as parental education. The classical Mincer setting includes controls for gender and regional dummies (not shown). *Source*: Authors’ calculations on EU-SILC 2011.

The relationship between preschool and wages in the last two specifications in Table 5 cannot be understood causally, because the models include one’s own education, which is itself influenced by preschool attendance (as shown in section 4.1 above). The models are thus misspecified; they suffer from bad control bias. We include them here, though, to show that even when controlling for educational attainment and thus looking at the preschool effect within educational classes, preschool has an economically and statistically significant effect on wages. While the raw difference in wages for people with and without preschool attendance could be understood as an upper bound of the preschool effect on wages (since we know that there is positive selection bias into preschool relative to wages), these specifications controlling for one’s own education provide a lower bound. These models eliminate the effect of preschool on wages which is mediated through educational attainment. We discuss this point further in section 4.2.1 below, as well as in Appendix D.

Looking more closely at the preschool effect on hourly wages, we study the impact of preschool attendance on wages for the sample of all employees in the data. In Table 6 we observe that attending preschool increases hourly wages by 7.1 percent, or 0.18 standard deviations of log hourly gross wages. The wage effect of preschool is smaller for those with more highly educated parents and stronger for migrants, though these effects are not statistically significant. The coefficient on the female dummy variable (not the interaction with preschool), which gives the gender wage gap, is estimated at 21%, which is similar to other estimates of the gender wage gap in Austria (Böheim et al. 2013). The finding that preschool attendance leads to a 7.1% increase in hourly wages is similar to, though a bit larger than, the findings from the UK (a 3.6% increase at age 33 and a 2.7% increase at age 42 (Goodman and Sianesi 2005)) and France (a 4.5% increase in hourly wages (Dumas and Lefranc 2010)). The stronger effects found for Austria may be because of the early tracking age, which makes preschool attendance that much more important in determining later education (track) and wages.

**Table 6.** Effects of preschool attendance on hourly gross wages.

	Est		Est		Est	
Preschool attendance	0.077	(0.015)	0.078	(0.017)	0.071	(0.019)
Intercept	2.619	(0.012)	2.618	(0.012)	2.626	(0.016)
Age			0.045	(0.007)	0.033	(0.015)
Age squared			−0.000	(0.000)	−0.000	(0.000)
Female			−0.205	(0.014)	−0.210	(0.022)
Father ed. 2			0.003	(0.017)	0.003	(0.025)
Father ed. 3			0.117	(0.026)	0.107	(0.056)
Father ed. 4			0.105	(0.037)	0.234	(0.105)
Mother ed. 2			0.079	(0.019)	0.068	(0.035)
Mother ed. 3			0.165	(0.023)	0.073	(0.053)
Mother ed. 4			0.154	(0.050)	0.183	(0.216)
Parent immigrant			−0.023	(0.057)	−0.049	(0.091)
PSAxAge					0.017	(0.018)
PSAxAge squared					−0.000	(0.000)
PSAxFemale					0.007	(0.028)
PSAxFather ed. 2					0.003	(0.034)
PSAxFather ed. 3					0.015	(0.064)
PSAxFather ed. 4					−0.146	(0.113)
PSAxMother ed. 2					0.017	(0.042)
PSAxMother ed. 3					0.117	(0.059)
PSAxMother ed. 4					−0.008	(0.222)
PSAxParent immigrant					0.036	(0.118)
Linear Controls			Yes		Yes	
Heterogenous TE						
N	2695		2695		2695	

*Notes*: This table shows the average treatment effect of preschool attendance (PSA) on gross hourly wages for employees. The bottom and top percentile of wage earners are dropped from the sample. Demeaned variables are used for all covariates and interactions. Regional dummies as well as dummies for the financial situation of the household and interactions were included as controls (not shown). *Source*: Authors’ calculations on EU-SILC 2011.

#### Mediation through educational attainment

One may ask to which extent the effect of preschool attendance on earnings is mediated through educational attainment. In other words, what portion of the wage increase from preschool attendance is channeled through the fact that preschool increases years of later schooling, which also increases wages? We use standard approaches from the literature on mediation analysis in, for example, VanderWeele (2015) or Pearl (2009), to answer this question. Details on the methodology can be found in Appendix C. Table 7 shows that the total effect of preschool attendance on earnings is estimated at about 8%, comparable with our model with linear controls (we use this specification here since the model does not allow for heterogenous treatment effects). The direct effect of preschool attendance on earnings is estimated at about 6%, which is close to the estimated effect we get from the Mincerian equation controlling for one’s later education. The average mediation effect is estimated at about 2%, which means that roughly 27% of the total effect of preschool on earnings is mediated through education. All effects are statistically significant, given the confidence sets which were estimated using 1000 simulations.

**Table 7.** Mediation of preschool attendance effect on hourly wages by years of schooling.

	Est	95% CI	
	Est	Conf low	Conf high
Average mediation effect	0.020	0.006	0.033
Direct effect	0.060	0.032	0.087
Total effect	0.080	0.050	0.110
% of tot eff mediated	0.254	0.183	0.408
N	2712	2712	2712

*Notes*: This table shows the average causal mediation effect of preschool attendance via years of schooling on log hourly wages for all workers based on 1000 simulations. *Source*: Authors’ calculations on EU-SILC 2011.

We now investigate the preschool effects beyond those for the individual, looking at the effect of preschool attendance on the probability that mothers work later in their children’s lives.

### Effects on mother’s labor force participation

Here we use the same methods as above to predict the effect of a respondent having attended preschool on the probability that their own mother worked when the respondent was 14 years old. We expect positive results, because having a child in preschool may allow the parents – and especially the mother, who is often the primary caregiver, particularly in Austria – the time and opportunity to participate in the labor force when the child is still young. The additional time at work when the child is young could help her later labor force participation, because the extra time at work enhances her human capital credentials and experience, along with connections in the labor force and opportunity for advancement.

Indeed, Table 8 shows that the mothers of children who went to preschool were 8.4 percentage points more likely to be working when the child was 14. This effect is tremendous, especially given the large gender gap in full time workers shown in Table 8. The results on the effect of preschool on mothers’ labor force participation are comparable to findings from studies looking at preschool effects in Canada (7.3 percentage point increase (Lefebvre and Merrigan 2008));; Israel (7 percentage point increase for Arab mothers (Schlosser 2005)); the US (a 7.5 percentage point increase for single mothers (Cascio 2009)); and Argentina (7–14 percentage points (Berlinski and Galiani 2007)).

**Table 8.** Effects of preschool attendance on the probability of the mother working at age 14.

	Est		Est		Est	
Preschool attendance	0.192	(0.013)	0.076	(0.015)	0.084	(0.017)
Intercept	−0.135	(0.010)	−0.062	(0.011)	−0.071	(0.014)
Age			−0.005	(0.007)	−0.007	(0.014)
Age squared			−0.000	(0.000)	0.000	(0.000)
Female			0.012	(0.013)	0.028	(0.021)
Father ed. 2			0.004	(0.016)	−0.014	(0.024)
Father ed. 3			−0.072	(0.025)	−0.095	(0.051)
Father ed. 4			−0.257	(0.035)	−0.412	(0.102)
Mother ed. 2			0.131	(0.017)	0.189	(0.032)
Mother ed. 3			0.185	(0.021)	0.196	(0.042)
Mother ed. 4			0.525	(0.056)	0.438	(0.155)
Parent immigrant			0.111	(0.050)	0.057	(0.084)
PSAxAge					0.009	(0.016)
PSAxAge squared					−0.000	(0.000)
PSAxFemale					−0.026	(0.027)
PSAxFather ed. 2					0.033	(0.032)
PSAxFather ed. 3					0.039	(0.059)
PSAxFather ed. 4					0.188	(0.110)
PSAxMother ed. 2					−0.089	(0.039)
PSAxMother ed. 3					−0.023	(0.049)
PSAxMother ed. 4					0.099	(0.169)
PSAxParent immigrant					0.107	(0.106)
Linear Controls			Yes		Yes	
Heterogenous TE						
N	5300		5300		5300	

*Notes*: This table shows the average treatment effect of preschool attendance (PSA) on the probability of the mother working when the respondent was 14. Demeaned variables are used for all covariates and interactions. Regional dummies as well as dummies for the financial situation of the household and interactions were included as controls (not shown). People who report being retired are excluded from the sample. *Source*: Authors’ calculations on EU-SILC 2011.

### Effects on the distribution of wages

**Figure 1.**
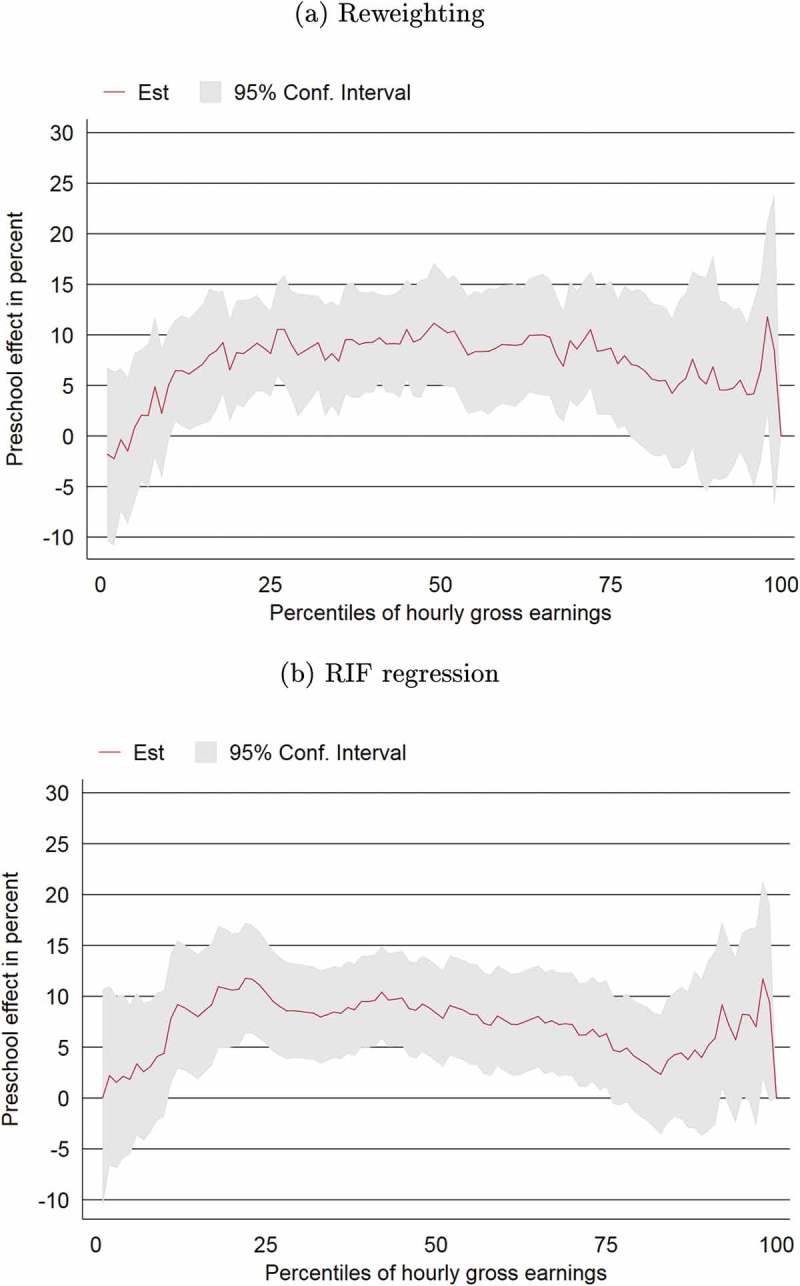
Effect of preschool attendance across the gross earnings distribution. *Notes*: Graph (a) shows the effect of preschool attendance on gross hourly wages across the full wage distribution using reweighting and (conditional) quantile regression. Graph (b) shows the effect of preschool attendance on gross hourly wages across the full wage distribution using recentered influence function regressions. The bottom and top one percentile of wage earners are dropped from the sample. *Source*: Authors’ calculations on EU-SILC 2011.

Finally, we turn our analysis to the effect of preschool attendance on the overall distribution of hourly wages. Figure 1 shows the effect of preschool across the wage distribution. The top graph is produced using reweighting with propensity scores, and is closer to a model with linear controls. The bottom graph is produced using re-centered influence function (RIF) regressions based again on a fully interacted model allowing for heterogenous treatment effects (see Appendix B for details on RIF regression). The patterns are very similar. At the lower end of the distribution of wages, the effect is relatively small. The small effect at the bottom of the distribution could be explained by the existence of minimum wages. Workers at this area of the distribution perhaps *would have* gained from preschool attendance, but the floor on their wages could mask the potential effects. In other words, preschool attendance may have given them a large percent increase in their (very low) wages, but since there is a minimum wage, the wage bump from preschool attendance is not observed (as it is, perhaps, subsumed in the bump up to earning minimum wages). At the upper end, the preschool effect also tends to be somewhat smaller. In between, the effect is rather stable between 5–10%. We conclude that the average effects estimated in our models are not driven by a small subset of the population but are rather stable across a large part of the distribution of wages. Across the bulk of the wage distribution, the preschool effect is about 10%.^7^ 7See Appendix D for a robustness check, in which we further account for one’s own education to measure the preschool effect within educational classes. The robustness check shows strong positive wage effects of preschool attendance, which should be a lower bound estimate for the overall effects.

Furthermore, Table 9 shows the effect of preschool attendance on three distributional measures. It shows that the effect on the Gini coefficient is economically and statistically not significant. At the bottom of the distribution, the finding that preschool nudges the ratio of wages at the $10^{th}$ percentile to those at the $50^{th}$ percentile downward – meaning that there is more inequality – is also statistically insignificant. As discussed above, this effect could be due to the presence of minimum wages, which guarantee that those without preschool at the lower end of the distribution already earn almost as much as those with preschool. The upper portion of the distribution (measured by the P90/P50 wage ratio), on the other hand, becomes more equal (economically but not statistically significant). Overall, the effects at the top and the bottom of the distribution cancel out, which one can see in the nonexistent effect on the Gini, which measures inequality in the middle of the distribution.

**Table 9.** Effect of preschool attendance on distributional measures.

	Est	Se.
Gini diff	−0.0009	0.0077
P10/P50 diff	−0.0333	0.0221
P90/P50 diff	−0.0610	0.0947

*Notes*: This table shows the average treatment effect of preschool attendance on distributional measures of the distribution of log hourly wages using reweighting. Standard errors are bootstrapped using 500 replicates. *Source*: Authors’ calculations on EU-SILC 2011.

Preschool attendance thus raises wages, in particular for workers in the middle of the wage distribution. To distill inequality at the lower end of the wage distribution, we would need other policy measures in addition to the minimum wages.

## V. Concluding remarks

In this paper, we used a rich data set from Austria to predict the effects of preschool attendance on later socioeconomic outcomes. We control for selection bias into preschool by using controls for parental education, region of residence, and the financial background of the household in which one grows up. While these controls may not address all of the mechanisms through while a child is selected into preschool, it is very feasible that they address the most important ones. Moreover, the institutional framework in Austria makes it unlikely that there are any financial constraints to sending children to preschool, which excludes an important source of selection bias from the outset. Using regressions with linear controls and allowing for heterogenous treatments effects, along with propensity score re-weighting, recentered influence function regressions, and mediation analysis, we show various ways in which preschool attendance has an important impact on economic life.

At the individual level, preschool leads to about two-fifths of a year more schooling once controlling for key background characteristics which influence selection into preschool, with more for women and second-generation migrants (the latter is not statistically significant). It also increases the probability of completing higher education by four to five percentage points, with lower effects for descendants of highly educated parents. The gross hourly wage effects of preschool attendance range from about seven to eight percent, and the effect of the probability of working full time is a positive six percentage points, with much stronger effects for women (an additional ten percentage points). The wage results for the whole population are strongest at the middle of the wage distribution (at about 10%), with a light equalizing effect coming from lower effects for higher earners. Lower earners are also less affected by preschool, perhaps because they are already protected by minimum wages. Finally, we find that mothers whose child attended preschool are eight percentage points more likely to be working when the child is 14 years old.

Consistent with the literature, the effects of preschool are overwhelmingly positive. Preschool attendance raises wages, educational attainment, and the labor market participation of the preschool attendee and his/her mother. Some states hesitate to implement more preschool programs because of additional costs. However, this analysis shows that preschool raises wages by about eight percent, and it increases labor force participation for the preschool attendees and their mothers. The increased income tax generated from the increased activity on the labor market and the higher wages could be used to help finance preschool programs. Indeed, as discussed by Kleven (2014), the use of taxes to subsidize goods and services which are complementary to labor market participation – such as preschool attendance, as we have seen in this paper – encourage and support active labor supply, which in turn brings money back into the system via income taxes. Interestingly, Berlinski, Galiani, and Manacorda (2008) shows that in Uruguay, the cost of expanding access to preschool (building the schools and paying the teachers) is well covered by the wage benefits enjoyed by those who get to attend preschool.

It is difficult to empirically disentangle the effects of preschool on later outcomes from the selection mechanisms which assign some people into preschool. We show how that in a case where external validity is the primary concern (as it is here, where we are interested in effects for the full population), a variety of methods can be employed to deal with potential selection bias. The raw gap in outcomes for those with and without preschool attendance is an upper bound estimate of the effect of preschool, but is ridden with (positive) selection bias. Our data used, from the 2011 Austrian EU-SILC, contain information on the education of the mother and the father, critical determinants of parental circumstances and thus the circumstances which influence the probability of a child being enrolled in preschool. Based on the fact that a large share of descendant’s outcomes are determined by parental education (Haveman and Wolfe 1995), we use measures of parental education as an important determinant of selection into preschool. In these models, we see consistently positive effects of preschool attendance. Going even further and accounting for one’s own education to measure the preschool effect within educational classes, which gives a lower bound estimate, shows strong positive wage effects of preschool attendance. We thus conclude that preschool has ubiquitously positive effects on the later economics outcomes studied here.

## Figures and Tables

**Figure 2.**
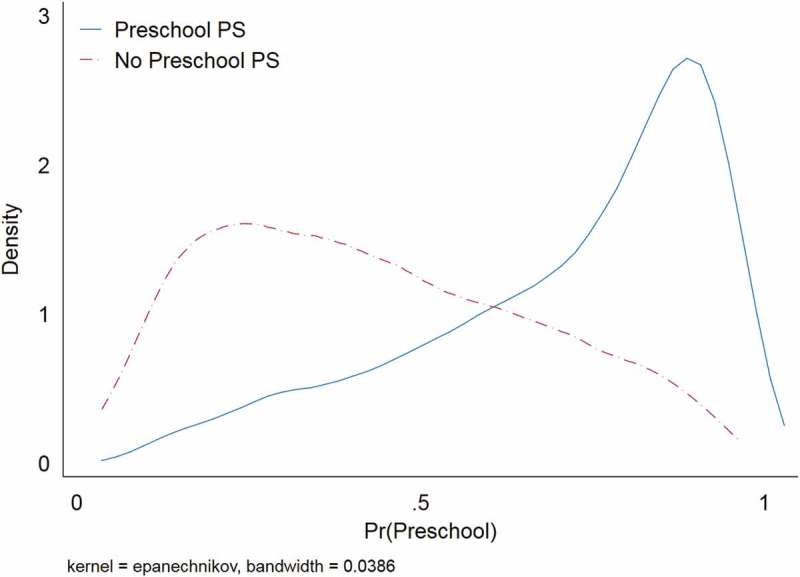
Propensity score for preschool attendance. *Notes*: This graph shows shows the distribution of propensity scores for preschool attendance and no preschool attendance based on a logit model using education of the mother, education of the father, age, age squared, second generation migrant, and regional dummies. *Source*: Authors’ calculations on EU-SILC 2011.

**Figure 3.**
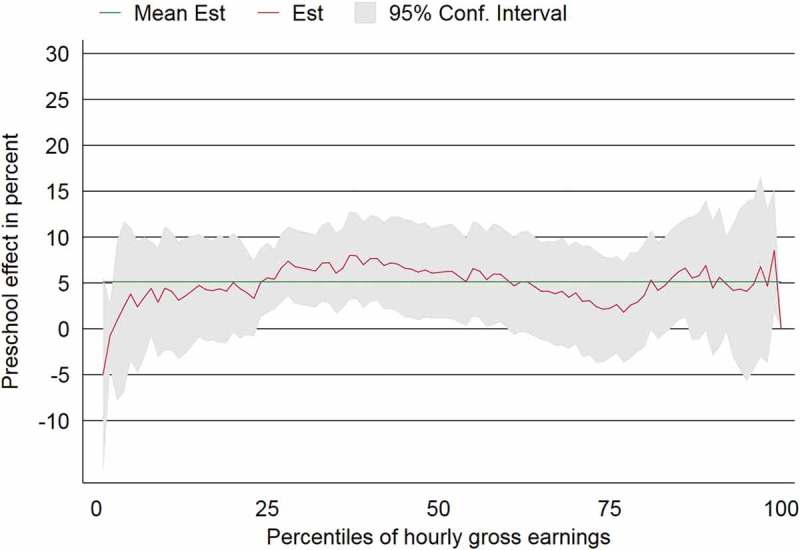
Preschool conditional difference across the gross earnings distribution including own education.

## Appendix A. Identification and discussion of identifying assumption

As in other studies that measure the returns to educational attainment, we are faced with potential selection bias in our estimates. We observe the differences in outcomes Y between those individuals i∈I who attended preschool $\left(T_{i}=1\right)$ and those who did not $\left(T_{i}=0\right)$, as shown in the left hand side of equation (4). These observed differences can be split up into two parts. First, there is the causal effect of preschool, which is what we are interested in measuring. Second, there is also potential selection bias, arising from the fact that potential outcomes under the condition of no preschool attendance ($Y_{0i}$) might be different for those who attend preschool and those who do not. This idea is expressed in the second expression on the right hand side of equation (4). Therefore, the observed differences in outcomes for those with versus those without preschool attendance may not be the effect of preschool. As we cannot observe the counterfactual outcomes $Y_{0i}$ for those who did attend preschool or $Y_{1i}$ for those who did not, we cannot empirically decompose the left into the right hand side of equation (4).

(4) $$\underset{Difference\;\;\;\;\;\;\;\;\;in\;\;\;\;\;\;\;\;\;\;\;\;\;Outcome}{\underbrace{E\left[Y_{i}|T_{i}=1\right]-E\left[Y_{i}|T_{i}=0\right]}}=\underset{Average\;\;\;\;\;\;\;\;effect\;\;\;\;\;of\;\;\;\;\;\;\;Pre-School}{\underbrace{E\left[Y_{1i}|T_{i}=1\right]-E\left[Y_{0i}T_{i}=1\right]}}\;\;\;\;\;\;\;\;\;\;\;\;+\underset{Selection\;\;\;\;\;\;\;Bias}{\underbrace{E\left[Y_{0i}|T_{i}=1\right]-E\left[Y_{0i}|T_{i}=0\right]}}$$

As in all observational studies,^8^ 8 A random assignment of preschool, which would solve the problem in the sense that $E\left[Y_{0i}|T_{i}=1\right]=E\left[Y_{0i}|T_{i}=0\right]$, is usually not feasible. we thus need to rely on assumptions about the assignment of preschool attendance in order to decompose the observed differences in economic outcomes into causal treatment effects and selection bias. The Conditional Independence Assumption (CIA) given in equation (5) is such an identification strategy. It states that once controlling for observable characteristics X, treatment assignment is random and selection bias disappears:

(5) $$\left\{Y_{0i},Y_{1i}\right\}⨿\;\;\;⏐T_{i}|X_{i}.$$

This assumption states that there is no (self-)selection into preschool correlated with potential economic outcomes, conditional on the covariates X. This assumption is not unproblematic, but reasonably credible with a rich set of covariates that determine selection into treatment, such as the ones we work with in this study. In our choice of covariates, we look for controls which ensure the CIA reasonably well without introducing bad control bias, which is another form of selection bias.^9^ 9 In our application, we actually only need the slightly weaker conditional mean independence assumption (CMI), which states that after controlling for X, the treatment does not affect the conditional mean of each potential outcome, whereas the conditional variance might depend on the treatment. Good controls are variables which are themselves not an outcome of preschool attendance; the background characteristics on which we want to control are strictly exogenous to preschool attendance (Imbens 2009).

To illustrate the credibility of our covariates in satisfying the CIA, we estimate a logit equation to obtain the probability of attending preschool conditional on X for all individuals in our sample. Figure 2 shows the resulting distributions of the probability of attending preschool (the propensity score (PS), as in Rosenbaum and Rubin (1983)), based on the covariates in X, for those who actually attended preschool (Preschool PS) and those who did not (No Preschool PS). One can clearly see the high predictive power of the selected covariates X: the people who attended preschool were indeed more likely to have attended, given their covariates, and the people who did not were less likely to have. The probabilities of the two groups are concentrated at the higher or lower levels of the probability distribution, corresponding to their actual attendance. Figure 2 also shows the large overlap, or common support, in the probability of attending preschool for these two groups. This large overlap illustrates that each individual *could* have received treatment or not (or, said differently, for each possible x∈X and each treatment state $T_{i}$, $0\left\langle Pr(T_{i}=i\right|x)<1$). It is the background characteristics, captured in X, which make them more or less likely to be assigned treatment.

To further study the credibility of the CIA in our empirical design, we present descriptive statistics of the covariates X used to model treatment assignment in table 7.^10^ 10 Note that there is missing information on the education of the father for 247 observations, on the education of the mother for 121 observations, on financial situation at age 14 for 114 observations and on the region of residence at age 14 for 41 observations, and leaving us (due to some overlap in the missing patterns) a maximum of 5,345 observations for the estimations. The table shows the means of the selection covariates in X for those who attended preschool and those who did not. People with and without preschool differ in some of their characteristics quite strongly; these groups differ in particular in their parental education, age, and region. The reweighted columns in Table 10 show that the characteristics of both subgroups balance rather well to the overall population once reweighted. We thus conclude that conditional on X, the treatment probability is almost equalized in the reweighted sample, giving ample support to the credibility of the CIA.

However, we cannot purge all selection bias from our estimates. We are confident, though, that this approach allows us to estimate reasonable effects for the full population and across the wage distribution. Using an IV approach exploiting some exogenous variation from a school reform, for instance, would help us to gain causal effects – but only for a subpopulation (some cohorts before and after such a reform) leading to better internal but rather bad external validity.

**Table 10.** Illustration of degree of rebalancing.

	Overall	No preschool	No preschool reweighted	Preschool	Preschool reweighted
Age	43.13	47.94	44.65	39.94	43.42
	(0.127)	(0.158)	(0.270)	(0.160)	(0.226)
Age squared	1,946.46	2,352.00	2,057.72	1,677.24	1,980.35
	(10.792)	(14.568)	(21.943)	(13.138)	(20.360)
Female	0.52	0.52	0.52	0.51	0.51
	(0.007)	(0.011)	(0.015)	(0.009)	(0.011)
Lower Secondary (Father)	0.45	0.37	0.46	0.51	0.46
	(0.007)	(0.010)	(0.015)	(0.009)	(0.010)
Upper Secondary (Father)	0.11	0.05	0.09	0.14	0.11
	(0.004)	(0.005)	(0.010)	(0.006)	(0.005)
Tertiary Edu (Father)	0.06	0.02	0.07	0.09	0.06
	(0.003)	(0.003)	(0.013)	(0.005)	(0.004)
Lower Secondary (Mother)	0.23	0.14	0.23	0.29	0.22
	(0.006)	(0.007)	(0.014)	(0.008)	(0.007)
Upper Secondary (Mother)	0.16	0.08	0.16	0.22	0.16
	(0.005)	(0.006)	(0.013)	(0.007)	(0.006)
Tertiary (Mother)	0.03	0.01	0.03	0.04	0.03
	(0.002)	(0.002)	(0.010)	(0.004)	(0.002)
Burgenland	0.04	0.03	0.04	0.05	0.04
	(0.003)	(0.004)	(0.006)	(0.004)	(0.003)
Carinthia	0.08	0.12	0.08	0.06	0.07
	(0.004)	(0.007)	(0.006)	(0.004)	(0.007)
Lower Austria	0.20	0.17	0.19	0.22	0.20
	(0.005)	(0.008)	(0.011)	(0.007)	(0.008)
Salzburg	0.07	0.07	0.07	0.07	0.07
	(0.003)	(0.006)	(0.007)	(0.004)	(0.006)
Styria	0.16	0.20	0.16	0.13	0.15
	(0.005)	(0.009)	(0.010)	(0.006)	(0.008)
Tirol	0.09	0.09	0.09	0.09	0.09
	(0.004)	(0.006)	(0.008)	(0.005)	(0.006)
Vorarlberg	0.04	0.02	0.04	0.06	0.04
	(0.003)	(0.003)	(0.007)	(0.004)	(0.003)
Vienna	0.12	0.07	0.14	0.16	0.13
	(0.004)	(0.006)	(0.013)	(0.006)	(0.006)
Second Generation Migrant	0.02	0.02	0.02	0.02	0.02
	(0.002)	(0.003)	(0.004)	(0.002)	(0.003)
Financial Background 2	0.10	0.13	0.10	0.07	0.09
	(0.004)	(0.007)	(0.007)	(0.005)	(0.007)
Financial Background 3	0.24	0.30	0.25	0.20	0.25
	(0.006)	(0.010)	(0.011)	(0.007)	(0.010)
Financial Background 4	0.32	0.28	0.32	0.35	0.32
	(0.006)	(0.010)	(0.014)	(0.008)	(0.009)
Financial Background 5	0.21	0.16	0.21	0.25	0.21
	(0.006)	(0.008)	(0.013)	(0.008)	(0.008)
Financial Background 6	0.06	0.02	0.06	0.08	0.06
	(0.003)	(0.003)	(0.011)	(0.005)	(0.004)

*Notes*: This table shows the means and reweighted means of the main variables used in the sample. Reweights are based on a logit estimation of the preschool attendance dummy on the set of covariates X. Using the propensity score, both subsets are then reweighted to the overall population. Standard errors are given in parentheses. *Source*: Authors’ calculations on EU-SILC 2011.

## Appendix B. Methods used to obtain effects beyond the mean

Aside from the preschool effects at the mean, we use two methods to investigate the preschool effects across the full distribution of the economic outcomes P(Y) and all their related measures ν(P(Y)) in section 4.4.

As discussed in section 2, we use the propensity score to balance the covariates of individuals with and without preschool attainment in order to construct counterfactual populations, as proposed in Rosenbaum and Rubin (1983) or DiNardo, Fortin, and Lemieux (1996). The counterfactual of interest is $P_{T=1}^{T=0}(Y)$, which is the distribution of the economic outcome for individuals without preschool attainment, with the same characteristics X as individuals with preschool attainment:

(6) $$P_{T=1}^{T=0}(Y):=\underset{X}{\int }P^{T=0}(Y,X)dP^{T=1}(X).$$

The counterfactual distribution in equation 6 can be rewritten as

(7) $$P_{T=1}^{T=0}(Y):=\underset{X}{\int }P^{T=0}(Y,X)\Psi_{X}(X)dP^{T=0}(X),$$

where the re-weighting function $\Psi_{X}$ is defined as

(8) $$\Psi_{X}:=\frac{P^{T=1}(X)}{P^{T=0}(X)}.$$

Reweighting requires the estimation of the ratio $\Psi_{X}$. We estimate the propensity of each individual to have preschool attainment using a logit model,

(9) $${\hat{P}}^{T=1}=Pr(T=1|X=x_{i})=\frac{1}{1+e^{-(\beta_{0}+X_{i}^{\prime }\beta )}},$$

and then reweight each individual i without preschool attendance by $1/\left(1-P^{T=1}(X)\right)$ and each individual with preschool attendance by $1/\left(P^{T=1}(X)\right)$ to create counterfactuals for the overall population without and with preschool attendance. The average treatment effect of preschool attainment on any economic outcome of interest ν is then defined as the difference between the reweighted counterfactuals:

(10) $$ATE=\nu (P_{rew}^{T=1}(Y))-\nu (P_{rew}^{T=0}(Y)).$$

As robustness check, we use more flexible re-centered influence function (RIF) regressions in section 4.4 to study the preschool effect across the distribution. A re-centered influence function is similar to a standard regression, except that the dependent variable is replaced by the recentered influence function of the statistic of interest (see Fortin, Lemieux, and Firpo 2009). Assume that ν can be written as the expectation of a function f of Y, ν=E[f(Y)]. The effect of the treatment on ν can be obtained from

(11) $$\nu^{T=1}-\nu^{T=0}=\int \left(E[f(Y)\left|X,T=1]-E[f(Y)\right|X,T=0]\right)dP(X).$$

In general, ν will not have this linear form but can be approximated by a linear first order expansion around $P^{*}$. This idea underlies the influence function regression approach proposed by Fortin, Lemieux, and Firpo (2009). Thus

(12) $$\nu (P)=\nu (P^{*})+\int IF(y;\nu ,P^{*})d(P-P^{*})(y)+R^{*},$$

where IF is the influence function of the parameter ν at $P^{*}$, and $R^{*}$ is a second order remainder term. Ignoring the remainder, this representation of ν has the linear form required for the use of the representation given in equation (11). In our case this linear approximation can be stated as

$$\nu (P)\approx \nu (P^{T=0}(Y))+\int IF(Y;\nu ,P^{T=0}(Y))dP(Y),$$

where $P^{T=0}(Y)$ is the baseline distribution of the economic outcome, which is in our case the distribution given no preschool attainment. Given the CIA and our treatment indicator $T_{i}$, we have

$$E[IF(Y)\left|X]=E[IF(Y)\right|X,T_{i}=i],$$

which justifies estimation of the following linear model again including all interactions of the treatment indicator with the covariate vector X, allowing for heterogenous treatment effects (like in equation 3 on page 7):

(13) $$E[IF(Y)|X,T_{i}]=X\cdot \beta^{0}+T_{i}\cdot X\cdot \beta^{1}.$$

The average treatment effect of preschool attainment $T_{i}$ on the population is thus given by

(14) $$ATE=E[X]\cdot \beta^{1}.$$

In the case of quantiles, the $IF(Y,Q_{\tau })$ is given as $\left(\tau -1\left\{Y\leq Q_{\tau }\right\}/f_{Y}(Q_{\tau }\right)$, where $1\left\{\cdot \right\}$ is an indicator function; $f_{Y}(\cdot )$ is the density of the marginal distribution of Y; and $Q_{\tau }$ is the population τ-quantile of the unconditional distribution of Y. The $RIF(Y;Q_{\tau })$ is then equal to $Q_{\tau }+IF(Y,Q_{\tau })$, and can be written as

(15) $$RIF(y;Q_{\tau })=Q_{\tau }+\frac{\tau -1\left\{y\leq Q_{\tau }\right\}}{f_{Y}(Q_{\tau })}=\frac{1\{y>Q_{\tau }\}}{f_{Y}(Q_{\tau }}+Q_{\tau }-\frac{1-\tau }{f_{Y}(Q_{\tau })}=c_{1,\tau }\cdot 1\{y>Q_{\tau }\}+c_{2,\tau }$$

where $c_{1,\tau }=1/f_{Y}(Q_{\tau })$ and $c_{2,\tau }=Q_{\tau }-c_{1,\tau }\cdot (1-\tau )$. As

$$E[1\{y>Q_{\tau }\}]=Pr(Y>Q_{\tau })=1-\tau ,$$

it follows that

$$E[RIF(y;Q_{\tau })]=c_{1,\tau }Pr(Y>Q_{\tau })+c_{2,\tau }=Q_{\tau }.$$

By the law of iterated expectations, we have

$$E[RIF(y;Q_{\tau })]=E_{X}\{E[RIF(y;Q_{\tau })]|X\},$$

and one can run a linear regression of the binary outcome variable $1\{y>Q_{\tau }\}$ on X (see Fortin, Lemieux, and Firpo (2011) and Fortin, Lemieux, and Firpo (2009)).

## Appendix C. Mediation Effects

In section 4.2.1 we introduce mediation effects, which separate the impact of preschool on earnings into (1) the direct effect (preschool → earnings) and (2) the effect which works through additional education (preschool → schooling → earnings). To calculate the mediation effect, one must run three basic regressions.

First, we regress outcome variable $Y_{i}$ (in this case earnings) on the preschool dummy $T_{i}$ and covariate vector $X_{i}$:

(16) $$Y_{i}=\alpha +\beta \cdot T_{i}+X_{i}\gamma +\varepsilon_{i}.$$

Second, we regress the mediator variable $M_{i}$, in our case years of schooling, on the preschool dummy $T_{i}$ and covariate vector $X_{i}$:

(17) $$M_{i}=\alpha +\beta \cdot T_{i}+X_{i}\gamma +\varepsilon_{i}.$$

Third, we regress the outcome variable $Y_{i}$ on the preschool dummy $T_{i}$, the mediator $M_{i}$, and the covariate vector $X_{i}$:

(18) $$Y_{i}=\alpha +\beta \cdot T_{i}+\tau \cdot M_{i}+X_{i}\gamma +\varepsilon_{i}.$$

Given equations 16, 17, and 18, $\hat{\beta }$ estimated from equation 16 is the total effect, while $\hat{\beta }$ of equation 17 times $\hat{\tau }$ of equation 18 is the mediation effect and the difference between the total effect and the mediated effect is the direct effect.

## Appendix D. Robustness checks

As a robustness check, we reproduce the top panel of Figure 1, showing the effect of preschool on gross wages across the distribution, in Figure 3. Here we include the respondent’s own education as a control variable, which leads to bad control bias since educational attainment is already an outcome of preschool attendance. However, this exercise illustrates that even inside similar educational groups, preschool attendance has a rather robust effect on earnings. When measuring the effect of preschool on wages while also controlling for one’s own education, we produce what could be interpreted as a lower bound of the causal effect of preschool.

*Notes*: This graph shows the effect of preschool attendance on gross hourly wages across the full wage distribution using reweighting. Full time employees only, including own education as an additional control variable. *Source*: Authors’ calculations on EU-SILC 2011.
